# Light-addressable potentiometric sensors: principles, strategies, and applications

**DOI:** 10.1039/d6cc02733d

**Published:** 2026-07-22

**Authors:** Yizhen Jia, Shulin Chen, Jinghua Li

**Affiliations:** a Department of Materials Science and Engineering, The Ohio State University Columbus OH 43210 USA li.11017@osu.edu; b Center for Brain Injury Recovery & Discovery, The Ohio State University Columbus OH 43210 USA; c Institute for Optical Science, The Ohio State University Columbus OH 43210 USA

## Abstract

Light-addressable potentiometric sensors (LAPS) provide a promising strategy for quantitative and spatially resolved detection of chemical biomarkers and electrophysiological signals in biological tissues. This review describes the working principles of LAPS, discusses key design considerations, and summarizes recent progress from the perspectives of semiconductor devices, sensing interfaces, and configurable illumination systems. Representative applications of LAPS in chemical imaging and electrophysiological detection are further reviewed, including pH mapping, ion detection, enzyme- and aptamer-based sensing, extracellular monitoring in microfluidic systems, and electrophysiological recordings from single cells, organoids, and *in vivo* tissues. Finally, current challenges and future directions are discussed to highlight the potential of LAPS for advanced biosensing and imaging applications. By comprehensively reviewing key aspects of LAPS and its applications, we aim to provide practical guidance for future biomedical research and global health initiatives.

## Abstract 2

Light-addressable potentiometric platform enables versatile, spatially resolved biosensing across chemical and electrophysiological targets.

## Graphical abstract

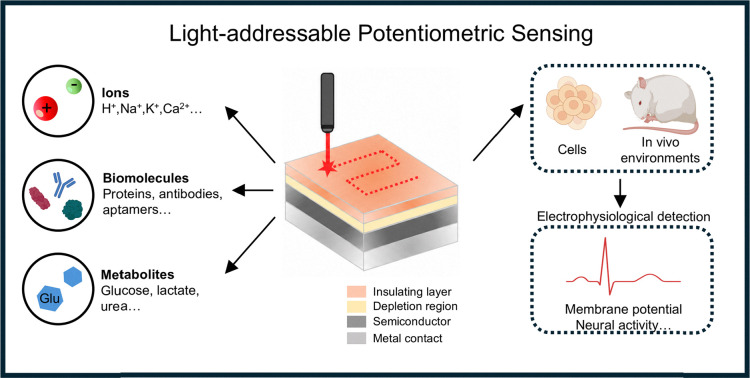

## 1. Introduction

Biofluids in the living systems contain a variety of chemical biomarkers that could reveal the health and age-based conditions relevant to biomedical research, advanced health care and clinical medicine.^1–6^ Interests in detection of biochemical signals in biofluids have motivated continued research to develop sensor technologies for continuous and quantitative health monitoring. In particular, imaging on a single entity within the surface of a soft tissue is of great importance for understanding bio-related heterogeneous processes. A record of the spatiotemporal distribution of biomarkers can provide valuable evidence enabling effective diagnosis and treatment of diseases. The capture, analysis and “mining” of such health data are crucial to both fundamental research and clinical practice.

However, the accurate quantification and mapping of chemical analytes in biotissues remain a challenging topic. Conventional techniques, such as bacteria culture, biopsies, microdialysis and ultraperformance liquid chromatography-tandem mass spectrometry (UPLC-MS/MS), have limited capability for imaging the distribution of biomarkers with a spatial resolution and/or are relatively time consuming. Similarly, nuclear magnetic resonance (NMR) or near-infrared diffuse reflectance spectroscopy require high cost, large-scale instrumentation with long data acquisition and processing times. Colorimetric sensing platforms with indicator dyes, on the other hand, suffer from the risk of dyes leaching out into biotissues (*e.g.*, non-healing wounds).^7^ Optical techniques based on fluorescent imaging and miniaturized microscopes have emerged as powerful tools;^8–11^ however, they still carry the drawback of requiring genetic modification. As a parallel approach, microelectrode^12,13^ and/or field-effect transistor arrays^14,15^ developed using microfabrication techniques can be used for addressable electrochemical measurements through potentiometric or amperometric sensing strategies. However, these arrays have individual sensing units with conductive pads, interconnects and dielectrics occupying the space. The predetermined geometry may limit the achievable density of effective working sites, making it challenging for high resolution imaging. Probe-based techniques can address this issue by recording local electrochemical properties of a contacted surface.^16^ However, the scanning speed is usually low and may introduce perturbation during measurement.

Collectively, these approaches face a common trade-off among spatial resolution, measurement flexibility, system complexity, and measurement perturbation. In many electrode- or transistor-array-based platforms, sensing sites are physically predefined by device layout, whereas probe-based techniques require mechanical scanning or contact. In contrast to these predefined or contact-based sensing configurations, LAPS uses a continuous electrolyte–insulator–semiconductor structure and defines the sensing location optically rather than lithographically. The working principle is as follows: A LAPS system consists of an unstructured electrolyte-insulator-semiconductor interface. Applying a voltage across the device biases the semiconductor towards depletion, and the modification in surface potential can be quantified by measuring changes in the photocurrent when the semiconductor is illuminated with a pulsed, focused laser beam (please see the following section for further details). The light-addressable nature of the platform eliminates the need for wires in conventional chemical sensors, enabling flexible site-specific addressing, real-time adaptability, and high-resolution imaging. Pioneering works on light-addressable sensors have been reported for biomedical investigations in as early as 1980s and recently.^17,18^ For example, molecular devices has commercially offered this technology as the cytosensor microphysiometer,^19^ designed to measure pH as an indicator of cellular activity. Beyond chemical sensing for crucial biomarkers (*e.g.*, pH, Na^+^, K^+^),^19–21^ LAPS has been also reported (although in a limited number of studies) to be capable of detecting electrophysiological activity in neurons and cardiac myocytes.^22,23^ Depending on the application, building an effective system typically requires close integration of optics, sensors, surface functional layers, and control systems.

Herein, we provide a summary of recent progress in LAPS with a focus on its application in the field of biosensing and bioelectronics. We begin with a brief overview of the system's working principle, followed by materials selection with a focus on enhancing spatial resolution and stability. The optics configuration is then discussed, providing the rationale for site-specific measurements and scalable light addressing. Surface functionalization strategies are reviewed in terms of improving sensitivity, selectivity, and efficient coupling with the semiconductor layer. Representative examples are presented to highlight the current state of the field, which can be broadly categorized into two main areas: chemical sensing and electrophysiological signal detection. Finally, we conclude with a summary and future outlook, aiming to provide guidance and reference for further research in this field.

## 2. Working principle of LAPS

**Fig. 1:**
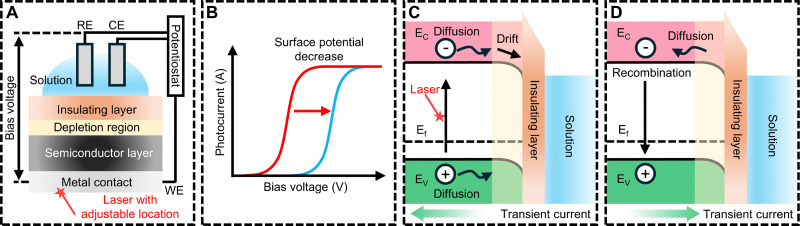
Schematic illustrations of LAPS working principle using a p-type semiconductor-based one as an example. (A) Schematic structure and depletion layer formation at the insulator–semiconductor interface under an applied bias. (B) Photocurrent–voltage characteristics of LAPS, showing a shift in the curve induced by changes in surface potential. (C) Energy band diagram under illumination, where light facilitates charge separation, then diffusion and drift effect creates a transient current. (D) Energy band diagram without illumination, where reversed diffusion effect and recombination create a reversed transient current.

Fig. 1A illustrates the basic structure of a LAPS device. The sensor consists of a semiconductor substrate covered by an insulating layer on the top surface and an electrical contact on the rear side, forming an electrolyte–insulator–semiconductor (EIS) system. In a typical measurement setup, a DC bias is applied between the reference and the working electrode (connected with metal contact) using, for example, an electrochemical workstation or potentiostat. Under modulated illumination, the resulting photocurrent is commonly read out directly or through a transimpedance amplifier or a lock-in amplifier. Upon application of a bias voltage (typically ± a few volts), a depletion layer forms at the insulator–semiconductor interface. The width of this depletion layer is controlled by the combined effect of the externally applied bias and the surface potential at the electrolyte–insulator interface.^24–26^ When the semiconductor is illuminated with modulated light, periodic photoexcitation generates an AC photocurrent response. Variations in the depletion layer thickness modulate its capacitance, which, together with the insulator capacitance *C*_i_, determines the measured current response. In a simplified equivalent circuit, the measured current can be expressed as:1
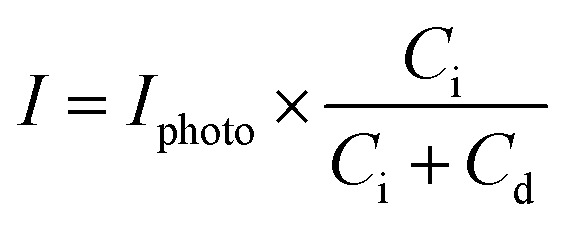
where *I* is the measured AC current signal, *C*_d_ is depletion region capacitance, *C*_i_ is insulator capacitance and *I*_photo_ is the photogenerated current induced by modulated illumination. This equation indicates that the fraction of the generated photocurrent collected as the LAPS signal is governed by the capacitive coupling between the insulator and depletion layer.^26^ As the depletion layer thickness increases (*C*_d_ decreases), a larger portion of the photocurrent is detected, whereas a thinner depletion layer reduces the measured signal.

Fig. 1B shows the corresponding photocurrent–voltage (*I*–*V*) characteristics. Variations in surface potential induce a lateral shift of the *I*–*V* curve along the voltage axis, which can be measured and used to determine the surface potential, and thereby the analyte concentration. This behavior can be understood from the relation:

2 *V*_effective_ = *V*_bias_ + *ψ*_surface_

where *V*_effective_ is the effective voltage across the EIS structure, *V*_bias_ is the externally applied DC bias between the reference and the working electrode, and *ψ*_surface_ is the surface potential at the electrolyte–insulator interface.^18^ The changes in surface potential effectively modify the total bias across the EIS structure, thereby shifting the bias voltage required to reach a given depletion condition.

Fig. 1C presents the energy band diagram under illumination, illustrated here using a p-type semiconductor as an example. Under illumination, photons with energies *hv* > *E* are absorbed by the semiconductor and generate electron–hole pairs locally within the illuminated region. This spatially confined photogeneration produces a nonuniform excess-carrier distribution and hence a carrier-concentration gradient, which drives photocarriers generated in the semiconductor bulk to diffuse toward the depletion region. Carriers generated within or reaching the depletion region are subsequently separated and transported in opposite directions by the space-charge electric field, producing a transient current. When modulated light with energy exceeding the bandgap irradiates the semiconductor, electron–hole pairs are generated. These photocarriers diffuse within the semiconductor bulk, and those reaching the depletion region are separated by the electric field, producing a transient current. As illumination continues, the transient current decays and approaches zero when the generation rate of carriers is balanced by recombination.^24^

Fig. 1D illustrates the condition when the light is turned off. In the absence of continuous photogeneration, charge separation ceases and excess carriers recombine. The redistribution of carriers leads to a transient current in the opposite direction. By periodically modulating the light, alternating transient currents create an AC photocurrent signal.^24^ This signal is typically detected using a lock-in amplifier with high modulation frequencies, achieving continuous readout of the LAPS response.

## 3. Device architectures of LAPS

**Fig. 2:**
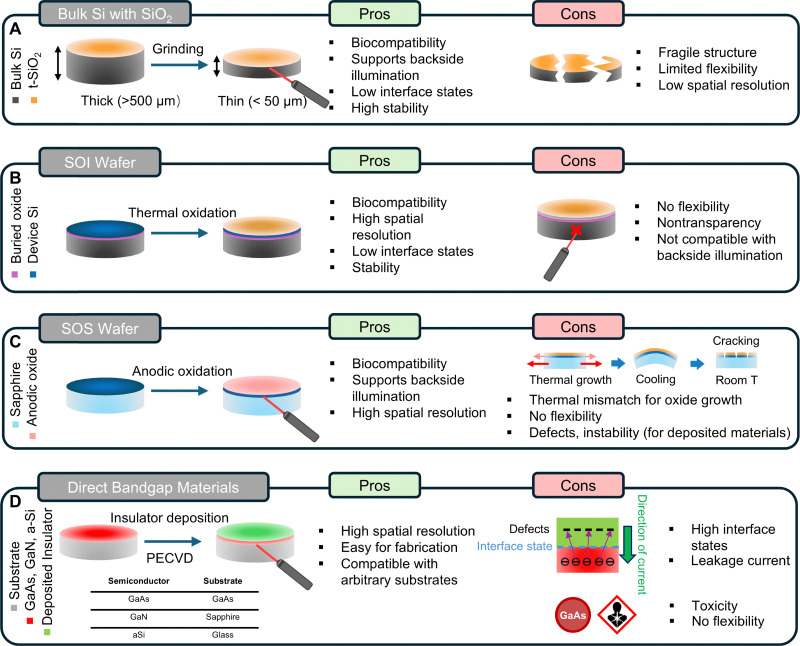
Schematic illustrations of fabrication strategies for LAPS devices based on different semiconductor materials, showing advantages and disadvantages of each method. (A) Conventional bulk Si-based LAPS with thermally grown SiO_2_ as the insulator. The substrate can be directly used for sensing. Alternatively, mechanical grinding can be used to thin substrate. (B) SOI wafer-based LAPS, with insulator layer formed by thermal oxidation. (C) SOS wafer-based LAPS, with insulator layer formed by anodic oxidation. (D) Direct bandgap materials-based LAPS for enhanced spatial resolution, with insulator layer formed by deposition techniques such as PECVD.

Following the basic operating principle described above, the next key consideration is device architecture, particularly the choice of semiconductor substrate and insulating structure, which influences the spatial resolution, stability, and practical applicability of LAPS. Early studies of LAPS primarily relied on thick, rigid single-crystalline silicon wafers as the semiconductor substrate due to their well-defined electronic properties, scalability, and compatibility with established microfabrication techniques. In the original device architecture, an EIS structure formed by growing a thermal SiO_2_ (*t*-SiO_2_) on the Si wafer (300–600 µm), often followed by a deposited Si_3_N_4_ or other pH-sensitive film such as Ta_2_O_5_ to create the sensing interface (Fig. 2A).^27–35^ However, the use of thick substrates limits the achievable spatial resolution in real-world practice: for biological applications, backside illumination is preferred, as the front side with thermal oxide contacts biofluids, cells, or tissues. When light generates charge carriers in the semiconductor at the back of the wafer, they must travel through the bulk Si to reach the space-charge region (depletion layer) at the insulator interface. During this process, lateral diffusion of carriers leads to spreading of the photocurrent signal and consequently reduced spatial resolution.^36^ Substrate thinning has therefore been explored to improve LAPS resolution. The most straightforward approach is mechanical grinding of the Si wafer, which can reduce the substrate thickness from several hundred micrometers to tens of micrometers, significantly suppressing carrier diffusion and enabling micrometer-scale imaging.^36–40^ In a representative high-resolution pH imaging study, reducing the silicon substrate thickness from 630 to 300 and 100 µm improved the resolvable line-and-space pattern from the millimeter/submillimeter range to 500 µm and then to 100–200 µm under 633 nm backside illumination. Further thinning to 20 µm, combined with 830 nm infrared illumination, enabled 10 µm line-and-space patterns to be resolved.^36^ However, grinding produces extremely thin and fragile wafers that are difficult to handle and integrate into practical devices.

To address the limitations of mechanically thinned Si substrates, increasing efforts have been devoted to developing LAPS structures with a variety of ultrathin semiconductors, including silicon on insulator (SOI), silicon on sapphire (SOS), and other direct-bandgap materials. In an SOI wafer, a thin device Si layer is separated from the bulk substrate by a buried oxide layer, which confines photogenerated carriers within a reduced thickness (Fig. 2B). A common practice is to grow another thermal SiO_2_ (*t*-SiO_2_) layer on top of the device Si layer as an insulator, like conventional bulk Si-based LAPS. The thin device layer suppresses the lateral diffusion of photogenerated carriers, thereby improving spatial resolution. T-SiO_2_ provides a high-quality interface due to the high-temperature oxidation process, ensuring biocompatibility, low interface state density, and long-term stability in biological environments. However, one unresolved issue remains to be that the rigid wafer structure limits mechanical flexibility. Furthermore, SOI is not compatible with back side illumination due to the nontransparency of the bulk substrate.^34^

On the other hand, SOS wafers use an optically transparent sapphire substrate to support a thin silicon layer, thereby enabling backside illumination (Fig. 2C). However, conventional thermal oxidation introduces significant challenges, as the mismatch in thermal expansion coefficients between silicon and sapphire generates stress during high-temperature processing. This stress consequently causes bending or even cracking of the thin silicon layer during cooling process. Low-temperature methods such as anodic oxidation therefore provide a more feasible strategy for forming the insulating layer. Anodic oxide can be grown in 0.1 M HCl by scanning the potential from 0 to 8 V *vs.* Ag/AgCl at a scan rate of 1 mV s^−1^, followed by holding at 8 V for 1 h.^41^ This anodic oxide preserves the thin semiconductor layer and supports high spatial resolution imaging. However, compared to thermally grown SiO_2_, anodic oxide exhibits higher defect density and increased leakage current, which lead to signal instability and reduced reproducibility. It also results in a higher number of interface states that can act as charge traps, leading to a decreased electrical performance. An 800 °C-annealing can increase the oxide quality but will again result in the thermal mismatch.^41^ To further improve interface properties, recent studies explore the replacement of oxide layers with self-assembled monolayers (SAMs) based on hydrosilylation as ultrathin insulating and functional interfaces. The SAMs directly graft onto silicon without an intervening silicon dioxide layer. The mild, highly efficient preparation processes enable better insulating effects, as demonstrated by electrochemical impedance measurements.^42^

Another strategy for improving sensitivity and spatial resolution employs direct bandgap materials, as the strong, shallow light absorption can confine photogenerated carriers near the illumination spot (Fig. 2D). Materials such as GaAs^43^ and GaN^44^ typically grow on lattice-matched or compatible substrates (*e.g.*, GaAs and sapphire) through epitaxy, while amorphous silicon (a-Si)^45–47^ can be directly deposited on substrates such as glass by plasma-enhanced chemical vapor deposition (PECVD), to form a semiconductor layer. These fabrication processes are simple while supporting high spatial resolution. However, similar to the issue discussed above, PECVD-deposited insulators such as Si_3_N_4_ for GaN^44^ and SiO_2_/Si_3_N_4_ double layers for a-Si,^47^ as well as anodic oxide such as SiO_2_ for GaAs^43^ on these semiconductor materials often exhibit relatively low quality, which introduces a large number of interface states at the semiconductor-insulator interface and leads to increased charge trapping and leakage current. In addition, the toxicity of some III-V materials (*e.g.*, GaAs) presents a significant challenge for biosensing applications. Finally, most existing technologies exploit wafer-based planar, bulky, and rigid structures, which limit the capability of imaging soft tissues. Overall, these limitations of LAPS technology have impeded its advancement toward bio-interfaces. Consequently, to date, LAPS has been primarily limited to simple *in vitro* experiments, with only a few studies reporting its use in *in vivo* settings. Further addressing them requires interdisciplinary insights that integrate fundamental semiconductor physics, optoelectronics, and engineering. Polar semiconductors including GaN and ZnO, have already been incorporated into LAPS.^44,48^ However, for practical LAPS devices, material selection is determined not only by carrier separation but also by scalability. In this respect, Si remains the most mature platform because wafer-scale processing and high-quality Si/SiO_2_-based interfaces enable reproducible and stable sensing areas. To the best of our knowledge, the specific contribution of crystal polarity and the associated polarization to LAPS performance has not yet been systematically investigated or quantified through controlled comparisons of opposite polarities or polar and nonpolar crystal orientations.

In general, Si-based LAPS provides stable and reproducible performance because high-quality oxide interfaces can be reliably formed through mature wafer-scale processing. These interfaces contribute to low leakage current, low noise, good uniformity, and improved reproducibility over large sensing areas. Direct bandgap semiconductors are advantageous for high-resolution LAPS because they enable more localized optical excitation and carrier generation. Moreover, the ultimate spatial resolution is jointly determined by optical absorption depth, carrier diffusion length, semiconductor thickness, illumination geometry, and device architecture. In addition, response time, long-term stability, and signal drift are usually case-dependent and can be influenced by the sensing-interface type, dielectric quality, fabrication parameters, electrolyte environment, and possible degradation of the semiconductor or interface.

## 4. Light addressing strategies

In addition to the material structure of the sensor, the illumination and scanning strategy is another key factor that determines the LAPS performance. The light addressing and scanning strategies for LAPS have evolved considerably as the technique has matured. Existing approaches can be broadly classified into three categories: motion-based illumination, digitally patterned illumination, and multi-point frequency-multiplexed illumination.

**Fig. 3:**
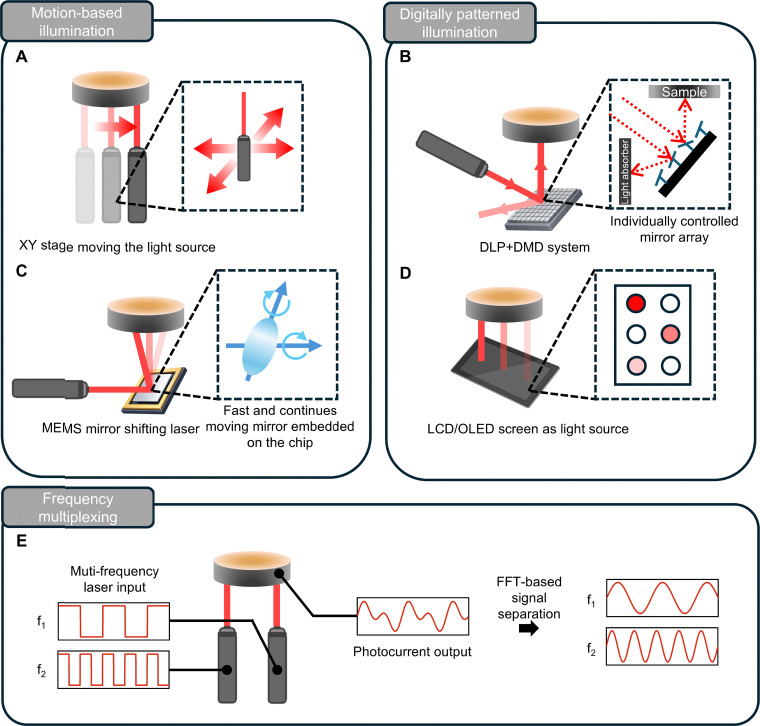
Illumination strategies for LAPS-based, spatially resolved sensing. (A) Motion-based illumination using an XY stage, where the light source is mechanically translated. (B) Illumination using MEMS mirrors, enabling fast and continuous beam steering with micromirror chip. (C) Digitally patterned illumination using a DLP system with a DMD, where programmable light patterns are projected onto the sensor surface without mechanical motion. (D) Illumination using pixelated light sources (*e.g.*, LCD/OLED screens), allowing direct generation of patterns with simple setup. (E) Frequency-division multiplexed illumination, where multiple light sources modulated at distinct frequencies are applied simultaneously, and the resulting photocurrent is separated in the frequency domain for parallel signal acquisition.

Motion-based approaches represent the most straightforward implementation, in which the illumination spot is sequentially moved across the sensing surface through controlled physical displacement of optical or mechanical components. In early implementations, scanning is achieved by mechanically translating the beam or the sample using an XY translation stage.^28^ For example, Fig. 3A demonstrates a scanning-laser LAPS system in which a focused (∼1 µm) modulated He–Ne laser is raster-scanned across the device to obtain two-dimensional pH images. The system employs either galvanometric mirrors for small-area scans or an XY stage for large-area scanning, achieving measurements over areas up to 48 mm × 48 mm, with typical scans of 64 × 64 pixels completed in approximately 7 min.

In addition to translation stages, laser scanning can also be achieved by using galvanometric scanners or micro-electromechanical system (MEMS) micromirrors, which enable rapid angular deflection of the illumination beam.^33,49^ As shown in Fig. 3B, MEMS mirrors utilize micrometer-scale reflective elements actuated to tilt and steer incident light. The mirror rotation is typically driven by electrostatic,^50,51^ electromagnetic,^52,53^ or piezoelectric actuation forces,^54^ allowing high-speed scanning with minimal mechanical displacement. For example, in a LAPS imaging system using MEMS micromirror to steer a modulated laser beam and perform raster scanning of the sensor surface, the authors demonstrate real-time chemical imaging at approximately 16 frames per second (fps), corresponding to a frame consisting of 10 × 8 pixels over a scanned region of about 2.8 mm × 5 mm. The system can also operate in a higher spatial resolution mode, in which chemical images with up to 200k pixels (500 × 400) are obtained over a 14.5 mm × 10.5 mm area within 40 s. These results demonstrate the advantages of MEMS mirrors in achieving high-speed LAPS imaging, along with flexibility in scanning speed and scanning patterns.

Compared with beam-steering approaches such as MEMS mirrors, digital light processing (DLP) systems provide a higher degree of flexibility in illumination control. By using a DLP system to control a digital micromirror device (DMD) consisting of thousands to millions of individually addressable mirrors, the reflected light can be precisely modulated to generate arbitrary and dynamically programmable illumination patterns (Fig. 3C).^55–57^ The use of a DLP system allows instantaneous reconfiguration of illumination patterns through software control, enabling rapid adaptation to different sensing regions without physical repositioning or scanning. Furthermore, independent control over the position, shape, and intensity of multiple light spots provides high flexibility for customized and dynamic measurement schemes. For example, in a compact DLP-based LAPS imaging system, the DMD chip provides a spatial resolution of 1024 × 768 mirrors, allowing the illumination to be projected onto the sensing surface with adjustable spot sizes and patterns.^55^ The system could address 480 × 320 measurement spots over a 20.8 mm × 15.6 mm sensing area, with adjustable spot sizes ranging from 434 µm down to 130 µm, demonstrating a better flexible spatial addressing capability compared with mechanically scanned systems. The flexibility of DLP-based illumination also facilitates the integration of LAPS with other optically driven functions. In another work, a DLP-based platform that integrates LAPS sensing with light-actuated AC electroosmosis (LACE) for particle manipulation in a microfluidic system is developed.^56^ In this setup, programmable illumination patterns generated by the DMD can be dynamically moved or reshaped through software control, allowing the same optical system to perform both sensing and manipulation functions. Such capability highlights the advantage of DLP illumination for multifunctional lab-on-chip platforms.

Another approach to patterned illumination is the use of display panels as light sources (Fig. 3D). In a miniaturized chemical imaging system, an organic light-emitting diode (OLED) display panel is used as the illumination source in replacement of conventional laser, optics, and mechanical scanning stage.^58^ The system employs a 20.3 mm × 13.5 mm (0.96-inch) OLED panel with a resolution of 96 × 64 pixels and demonstrates different illumination patterns. By eliminating the complex optical setup, this configuration significantly simplifies the instrumentation and enables a compact imaging platform, paving the way for the development of miniaturized and integrated LAPS devices. However, compared with DMD based systems where unwanted light can be directed to a dedicated optical absorber, display panel-based illumination may exhibit lower contrast between illuminated and dark regions due to residual light leakage and background illumination, which can lead to a reduced signal-to-noise ratio in photocurrent measurements.^55^

Finally, frequency multiplexing provides the key to fully utilizing the flexibility offered by advanced illumination systems. In frequency division multiplexed (FDM) LAPS, multiple light spots are modulated at different frequencies and illuminate the sensor simultaneously (Fig. 3E).^59,60^ The resulting photocurrent is a combination of all frequency components, and the signal from each illumination point can be separated using Fourier analysis, thus achieving simultaneous measurement at multiple locations. Using this concept, a high-speed chemical imaging system based on front-side illuminated LAPS combined with a light-emitting diode (LED) matrix is developed.^59^ In this system, five LEDs were modulated at different frequencies (6–10 kHz) to enable frequency-division multiplexed measurements. By sequentially switching the illuminated rows of a 7 × 5 LED matrix, the system scanned 35 measurement points across the sensing surface, achieving chemical imaging of pH distributions at frame rates of up to 70 frames per second. Table 1 summarizes the representative laser and illumination parameters used in these LAPS imaging systems.

**Table 1:** Representative laser and illumination parameters used in LAPS imaging systems

Light source	Scanning method	FDM (channels)	Speed (per spot)	Speed (per image)	Area (mm × mm)	Pixels	Spot size	Ref.
He–Ne laser (633 nm)	XY translation stage	No	110 ms	30 min	6 × 6	128 × 128	1 µm	28
Laser (633 nm)	Analog MEMS micromirror	No	0.75 ms	60 ms	2.8 × 5	10 × 8	—	33
LED (from DLP projector)	DLP (DMD micromirror array)	No	0.4 ms (high resolution)	2–60 s	20.8 × 15.6	480 × 320	434 × 434 to 130 × 130 µm	55
LED (from DLP projector)	DLP (DMD micromirror array)	No	200 ms	5 s	40 × 26	480 × 320	—	57
OLED display	OLED display	No	—	—	20 × 13	96 × 64	Pixel-defined (∼100–300 µm scale)	58
LED matrix	LED array + FDM	5	2 ms	70 ms	36 × 36	7 × 5	—	59
LED matrix	LED array + FDM	16	6.25 ms	100 ms	15 × 15	4 × 4	2000 µm	60

## 5. Surface functionalization strategies

LAPS has been widely used for the detection of diverse chemical and biological targets, including ions, proteins, and nucleic acids based on the potentiometric sensing mechanism.^61–64^ Recent advances in potentiometric sensing interfaces also provide useful context for LAPS functionalization. In solid-contact ion-selective electrodes (SC-ISEs), the liquid internal contact is replaced by solid ion-to-electron transducers, enabling miniaturization, chip integration, portability, and improved potential stability for on-site, wearable, and complex-sample analysis.^65^ In parallel, biocompatible polymeric sensing membranes, especially silicone-based ion-selective membranes, have attracted interest because of their low water uptake, long lifetime, and improved biocompatibility compared with conventional plasticized poly(vinyl chloride) (PVC) membranes.^66–68^ Molecularly imprinted polymer (MIP)-based potentiometric sensors represent another important direction, where synthetic recognition sites expand potentiometric sensing from inorganic ions toward organic and biological targets while offering advantages such as chemical stability, low cost, and easier preparation compared with many biological receptors.^69–71^ Although these potentiometric platforms differ from LAPS in device architecture, they share the general principle of converting interfacial potential changes into electrical signals. The uniqueness of LAPS remains its light-addressable operation. However, advances in SC-ISEs, biocompatible membrane matrices, and MIP-based molecular recognition and other potentiometric sensors, may be used for future LAPS surface functionalization and the development of more robust, miniaturized, and flexible light-addressable systems. The sensing capability of LAPS can be tailored by introducing different sensing layers and surface functionalization strategies on the insulator surface. In this section, we categorize these approaches based on their sensing materials and functionalization strategies to illustrate how common targets are detected using LAPS.

**Fig. 4:**
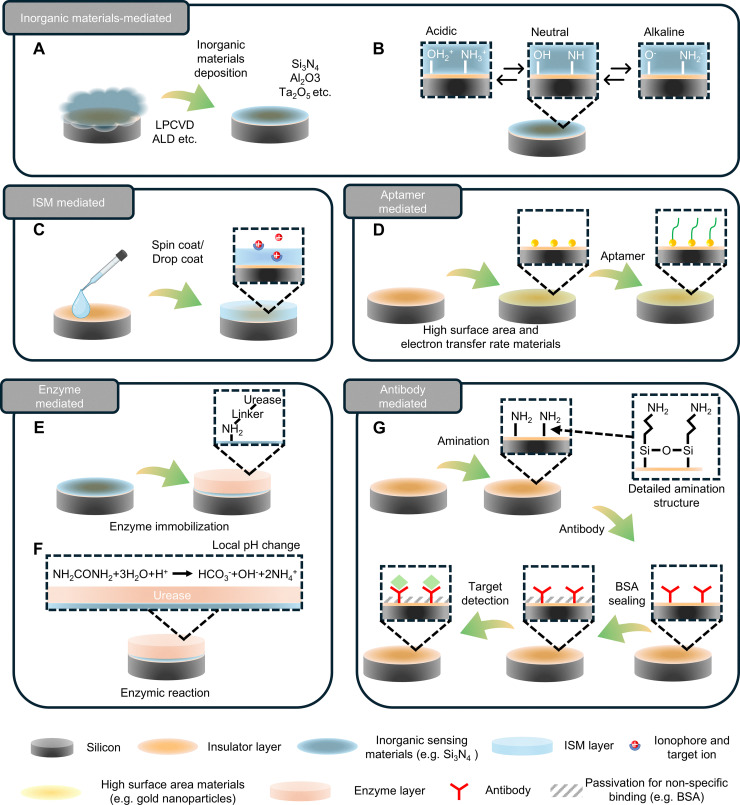
Surface functionalization strategies for LAPS-based biochemical sensing. (A) Inorganic material-mediated sensing (mainly used for pH sensing), where layers (*e.g.*, Si_3_N_4_, Al_2_O_3_, Ta_2_O_5_) are deposited by techniques such as LPCVD. (B) pH sensing mechanism of Si_3_N_4_ as an example. (C) Ion-selective membrane mediated sensing, where membranes are directly deposited on the surface of the insulator layer. (D) Aptamer-mediated sensing, where aptamers are immobilized by mediating layer and chemical bonds. (E) Enzyme-mediated functionalization, involving immobilization of enzymes on a pH sensing layer. (F) Enzymatic sensing mechanism, where biochemical reactions (*e.g.*, urea hydrolysis) induce local pH changes that are transduced by the LAPS device. (G) Antibody-mediated sensing, where surface functionalization (*e.g.*, amination) enables antibody immobilization, followed by target binding and blocking (*e.g.*, BSA) to reduce non-specific interactions.

One class of sensing interfaces relies on inorganic pH-sensitive materials deposited on the semiconductor surface, as illustrated in Fig. 4. In this approach, a pH-responsive layer such as silicon nitride (Si_3_N_4_) is deposited on the insulator substrate.^28,31,33,37,61,72,73^ The Si_3_N_4_ sensing layer provides a proton-sensitive interface that enables detection of pH variations through changes in the surface potential. Surface groups such as OH and NH_2_ groups undergo protonation or deprotonation depending on the solution pH, resulting in changes in surface charge density and surface potential. This variation then changes the thickness of the depletion layer in the underlying semiconductor and leads to a measurable shift in the photocurrent signal. In addition to Si_3_N_4_, other materials such as Ta_2_O_5_,^74,75^ Al_2_O_3_,^76^ HfO_2_^77,78^ and NbO_*x*_^79^ have also been investigated as pH-sensitive layers. Pulsed laser deposition (PLD) can form Ta_2_O_5_ or Al_2_O_3_ film on Si substrate as the sensing interface, while sputtering has been widely used for the fabrication of HfO_2_ and NbO_*x*_ sensing layers. The pH response of LAPS originates from changes in the electrolyte–insulator interfacial potential and is generally governed by Nernstian/site-binding behavior. Therefore, properly processed pH-sensitive layers often show comparable near-Nernstian sensitivities.^80^ In contrast, stability is more strongly dependent on the specific deposition method and parameters. In general, inorganic materials typically feature better stability than organic materials.

Another functionalization strategy involves ion-selective membranes (ISMs) deposited on the sensing surface. These membranes contain ionophores that selectively bind specific ions and generate a phase boundary potential according to the Nernst equation. The resulting potential change modulates the surface potential of the underlying LAPS structure and can be detected through shifts in the photocurrent signal. Ionophores can be designed for targets including K^+^, Ca^2+^, Mg^2+^, and Li^+^.^81–86^ The membrane can be conveniently applied to the sensor surface through spin coating or drop casting to form the sensing layer.

Aptamer-based sensing interfaces have also been integrated with LAPS for selective detection of biomolecules.^62,63,87^ Aptamers are short single-stranded nucleic acids that can bind target molecules with high specificity through structural recognition. Surface functionalization of aptamer-based LAPS sensors typically involves introducing bonding for immobilization of the aptamer probes. For example, gold nanoparticles can be attached to the insulation layer with (3-mercaptopropyl)triethoxysilane (MPTES) modifications.^62^ The gold nanoparticles provide a high-surface-area interface for probe immobilization. Thiolated aptamers are then attached to the Au surface to form the sensing layer. Upon binding of the target to the immobilized aptamer probes, the surface charge at the sensing interface changes, leading to a shift in the surface potential that can be detected through shifts in the photocurrent–voltage characteristics of the LAPS device. As opposed to using materials like gold nanoparticles as a mediator, treatment of the sensor surface with 3-aminopropyltriethoxysilane (APTES) solution can introduce amine groups, therefore achieving the immobilization of aptamers.^63^ Together, these studies demonstrate different strategies for immobilizing aptamer probes on LAPS surfaces. Beyond immobilization, reusability and long-term stability are also important for aptamer-functionalized LAPS, especially for repeated or continuous biosensing. Aptamer-based sensors can be regenerated using various physical and chemical approaches, including light-triggered conformational switching,^88^ thermal denaturation,^89^ pH adjustment,^90^ and rinsing with chemicals,^91^ to dissociate and remove bound target molecules. However, corresponding studies on aptamer-functionalized LAPS remain limited. Moreover, regeneration procedures may affect the LAPS response because changes in pH and ionic strength directly alter the electrolyte–insulator interfacial potential. Future studies should therefore develop mild, LAPS-compatible regeneration protocols and systematically evaluate regeneration efficiency and long-term storage stability.

Enzyme-based sensing in LAPS is typically achieved by immobilizing an enzyme layer on top of a pH-sensitive sensing surface, allowing biochemical reactions occurring at the interface to be detected through local pH variations.^64,92–94^ Take urea sensing LAPS as an example, the urease catalyzes the hydrolysis of urea, producing ammonium and hydroxide ions and leading to an increase in local pH. The resulting pH change modulates the surface potential of the pH sensing LAPS device and allows the concentration of urea to be indirectly detected. In addition to pH-mediated detection, some enzymatic reactions can also be directly monitored through potentiometric responses at the sensing surface. For example, the enzymatic oxidation of glucose generates hydrogen peroxide as a reaction product, which induces a change in the surface potential at the sensing interface and therefore be directly detected by recording shifts in the photocurrent–voltage characteristics of the LAPS device.^95^

Antibody-based sensing can also be implemented with LAPS for label-free immunodetection.^96–98^ In a LAPS biosensor for immunoglobulin G (IgG) detection, the silicon wafer is first silanized to introduce surface amine groups, followed by glutaraldehyde crosslinking to enable covalent attachment of IgG antibodies.^99^ After antibody immobilization, bovine serum albumin (BSA) is applied to block remaining active sites and reduce non-specific adsorption. The functionalized surface can then specifically capture IgG antigens, and the resulting antigen–antibody binding altered the interfacial potential, and therefore be measured by photocurrent change. Table 2 summarizes the representative studies discussed in this section, covering the target species, sensing interfaces, demonstrated applications, and laser systems used in LAPS-based chemical and biosensing.

**Table 2:** Summary of representative LAPS studies for chemical and biosensing applications

Year	Target	Sensing material	Sensitivity	Analytical range	Operating condition	Application demonstrated	Laser system	Limitations	Ref.
2019	Extracellular pH	Si_3_N_4_	51.18 mV dec^−1^	pH 4.4–10.4 calibration	Phosphate-buffered saline (PBS) calibration; Dulbecco's Modified Eagles's Medium (DMEM)	Real-time extracellular acidification monitoring; cell metabolism analysis under glucose and drug (doxorubicin) stimulation in microfluidic system	Laser diode (450 nm)	Bubbles cause interference in measurement	61
2020	In vivo pH (brain extracellular pH)	Si_3_N_4_	57.5 ± 2.2 mV dec^−1^	pH 4–7 calibration	PBS for pseudo-reference OCP; standard pH buffers; *in vivo* rat hippocampus	Spatially resolved, real-time *in vivo* brain pH monitoring	Laser diode (451 nm, *via* multimodal fiber)	Pseudo-reference stability and Cl^−^ interference require careful calibration	72
1994	pH (H^+^ distribution)	Si_3_N_4_	56 mV dec^−1^	—	pH buffer calibration; yeast colonies on PGY agar	2D pH imaging of chemical gradients and biological samples (yeast colonies)	He–Ne laser (633 nm), scanning *via* galvanometric mirrors/XY stage	Spatial resolution is limited by photocarrier diffusion and wafer thickness	28
1994	pH (H^+^ distribution)	Si_3_N_4_	—	—	Standard electrolyte of pH 6.86	pH imaging and spatial resolution improvement *via* Si thinning (down to 100 µm)	He–Ne laser (633 nm), galvo mirrors	Thinning involves resolution/robustness trade-offs	37
2014	pH (H^+^ distribution)	Si_3_N_4_	—	—	pH buffers; 0.1 M HCl injection into 0.1 M KCl or pH 7 buffer	Real time pH imaging of acid injected into solution	Red laser, microelectromechanical system	High-speed scanning reduces signal/noise ratio	33
2014	pH (H^+^ distribution)	Si_3_N_4_	—	—	Phosphate buffer; 10 mM HCl/NaOH injection; PDMS microfluidic channel	Real time pH imaging in microfluidic	LED and optical fiber	Response/recovery behavior depends on injection volume and flow conditions	1
2001	pH; penicillin *via* pH change	Ta_2_O_5_; porous Si with enzyme	57.5 mV dec^−1^	pH 3–10; penicillin 250 µM–10 mM	Standard pH buffers	pH sensing; enzyme-based detection of penicillin	Modulated light	Indirect enzymatic pH readout limited by enzyme stability	74
2000	pH (H^+^ detection)	Al_2_O_3_	57 mV dec^−1^	pH 2–11.3	pH buffers	pH sensing	IR laser (830 nm)	Validation in complex application environment is not reported	76
2010	Na^+^, also pH	HfO_2_	Na^+^: 31.8 mV dec^−1^; pH: 52.2 mV dec^−1^	Na^+^: 10^−5^–10^−1^ M; pH 2–12	5 mM Tris/HCl, pH ∼8.4 for Na^+^; commercial pH buffers	Sodium ion detection	IR LED array	Validation in complex application environment is not reported	77
2013	NH_4_^+^	HfO_2_	37 mV dec^−1^	10^−5^–10^−1^ M	5 mM Tris-HCl, pH 8.0	NH_4_^+^ detection	Near-infrared LED (890 nm)	Requires rapid thermal annealing/CF_4_ treatment	78
2015	pH	NbO_*x*_	57–60 mV dec^−1^	pH 2–12	pH buffers	pH imaging	Red laser (∼658 nm), X–Y stage scanning	Drift/hysteresis and composition control require further study	79
1999	K^+^, Ca^2+^, Mg^2+^	Al_2_O_3_, ion-selective membranes	K^+^: 50 mV dec^−1^; Ca^2+^: 16–25 mV dec^−1^; Mg^2+^: 19 mV dec^−1^	K^+^: 10^−5^–0.1 M; Ca^2+^: 10^−4^–0.1 M; Mg^2+^: 10^−4^–0.1 M	20 mM Tris/HCl, pH 7.0	Ion-selective detection of multiple cations	—	Validation in complex application environment is not reported	81
2001	H^+^, Na^+^, K^+^, Ca^2+^	Si_3_N_4_, ion-selective membranes	H^+^: 70 mV/pH; K^+^: 57 mV dec^−1^; Ca^2+^: 35 mV dec^−1^	—	RPMI 1640-based culture medium	Multi-ion, real-time monitoring of extracellular ion dynamics in living cells	LED array (900 nm), frequency-division multiplexing	Membrane cross-interference is not fully considered, and detailed signal studies are needed.	82
2003	K^+^, Ca^2+^	Si_3_N_4_, ion-selective membranes	K^+^: 57 mV dec^−1^; Ca^2+^: 27–28 mV dec^−1^	K^+^: 10^−5^–10^−1^ M, LOD 2 × 10^−6^ M; Ca^2+^: 10^−5^–10^−1^ M, LOD 5 × 10^−6^ M	—	Simultaneous multi-ion detection	IR laser beam	pH/cross-ion interference and daily calibration affect use	83
2005	Li^+^, K^+^, Ca^2+^, Mg^2+^, pH	Si_3_N_4_, ion-selective membranes	Monovalent ions: 57–59 mV dec^−1^; divalent ions: 26–27 mV dec^−1^	—	—	Multi-ion sensing and chemical imaging	A scanning laser beam or an array of LEDs	Multianalyte measurements can suffer from cross-sensitivity	84
2024	Ca^2+^	ion-selective membranes	Constant-voltage mode: 93.65 nA dec^−1^; constant-current mode: 21.77 mV dec^−1^	Ca^2+^: 10^−7^–10^−2^ M; LOD 100 nM	0.1 M CH_3_COOLi background; human serum and urine	Dynamic monitoring of Ca^2+^ in solution; demonstrated in human serum and urine	Laser diode (520 nm)	Label-free Ca^2+^ imaging and *in vivo* validation remain future directions.	85
2012	ATP	DNA aptamer	ATP: 9 mV µM^−1^	ATP: 10^−8^–10^−4^ M; LOD 2.6 × 10^−8^ M	PBS/Tyrode's solution; single taste receptor cells	Real-time monitoring of ATP secretion from single taste receptor cells	He–Ne laser (543.5 nm)	Regeneration is limited to about five cycles before signal decreases	87
2020	Alpha-fetoprotein	AFP aptamer	AFP: 2.5892 mV µg^−1^ mL^−1^	AFP: 0.1–100 µg mL^−1^; LOD 92.0 ng mL^−1^	PBS, 0.2 M, pH 6.5; human serum	Label-free detection of AFP; clinical biomarker sensing in serum	Laser diode (650 nm)	Stability is limited (75% signal after 7 days)	62
2026	Adenosine	DNA aptamer	—	Adenosine: 0.05–100 nM; LOD 0.01 nM	PBS, 10 mM, pH 7.5; 10-fold diluted human serum	detection of adenosine, validated in human serum	He–Ne laser (543.5 nm)	Regeneration is not demonstrated	63
1996	Urea	Si_3_N_4_ + urease	—	Urea: 10^−7^–10^−2^ g mL^−1^	—	2D chemical imaging of urea	IR laser (830 nm)	Indirect enzymatic pH readout saturates at high urea concentrations	92
2011	Urea	Si_3_N_4_ + urease	—	Urea: 0.3 × 10^−3^–10^−1^ M	PBS	Real-time urea detection in flow channel	Modulated IR laser (830 nm), dual-beam FDM	Single-channel readout suffers from drift and flow-related fluctuations	64
2013	Acetylcholine	Ta_2_O_5_ + enzyme membrane	—	ACh lower limit ∼0.1 mM; valid response up to ∼5 mM	Phosphate-buffer solution	ACh imaging; real-time spatial mapping of neurotransmitter dynamics	IR LED array	Resolution is not sufficient for single-cell release	93
2020	Glucose	Glucose oxidase	Glucose: 101.1 mV dec^−1^	Glucose: 0.01–100 mM; LOD 0.003 mM; LOQ 0.01 mM	0.1 M PBS, pH ∼7.0; diluted blood and urine; NIST human serum	Glucose determination in blood and urine	Optical-fiber-coupled laser module (680 nm)	Enzymatic readout is limited by enzyme stability	95
2020	Extracellular pH	Ta_2_O_5_	pH: 57 ± 1.6 mV dec^−1^	pH 5–9	Titrisol pH buffers; CHO-K1 cells	real-time imaging of extracellular acidification of living cells in microfluidic channels	Laser diode (∼780 nm)	O_2_ plasma improves cytocompatibility but can degrade LAPS *via* ultraviolet-induced dielectric defects	75
2025	Ca^2+^	Ion-selective membranes	Ca^2+^: 116.4 nA dec^−1^	Linear range: 10^−1^–10^−5^ M	0.1 M CH_3_COOLi background; HL-1 cell culture medium	Real-time extracellular Ca^2+^ monitoring and imaging in 2D & 3D HL-1 cells; calcium channel drug evaluation; spatially resolved Ca^2+^ imaging	Laser diode (520 nm)	Drug background influence measurements	86
2024	pH	Si_3_N_4_ modified with PS colloidal spherical lens array	pH: 57.38/58.59 mV dec^−1^	pH 3–8	pH buffers	High spatiotemporal pH imaging	Modulated laser	Validation is mainly limited to pH buffers and controlled urease reaction imaging	73

In addition, recent progress in broader potentiometric sensing also provides useful design concepts for LAPS functional interfaces, although these developments should be distinguished from LAPS itself. For example, in solid-contact potentiometric sensors, liquid internal contacts are replaced by ion-to-electron transducing layers to avoid problems associated with conventional inner filling solutions, including evaporation, pressure sensitivity, osmotic effects, limited miniaturization, and maintenance requirements, thereby enabling miniaturized, low-maintenance, and wearable formats.^65,100^ Nanostructured transducers, such as carbon nanotubes, have been widely explored to improve charge-transfer efficiency, interfacial capacitance, potential stability, and device miniaturization.^101–103^ In addition, molecularly imprinted polymers (MIP) have been used as synthetic recognition layers for selective potentiometric detection of molecular targets.^70,101^ For example, a recent miniaturized screen-printed potentiometric electrode combined single-walled carbon nanotubes as the solid-contact transducer with an MIP-based recognition layer for lidocaine detection. Although these studies are not direct LAPS demonstration, such advances in solid-contact transduction, nanostructured interfaces, and MIP recognition may inform future LAPS interface design.

## 6. Applications of LAPS in chemical imaging

Building on these surface functionalization strategies, LAPS has been applied to a variety of chemical imaging scenarios, providing spatially resolved and label-free detection of interfacial chemical changes.

**Fig. 5:**
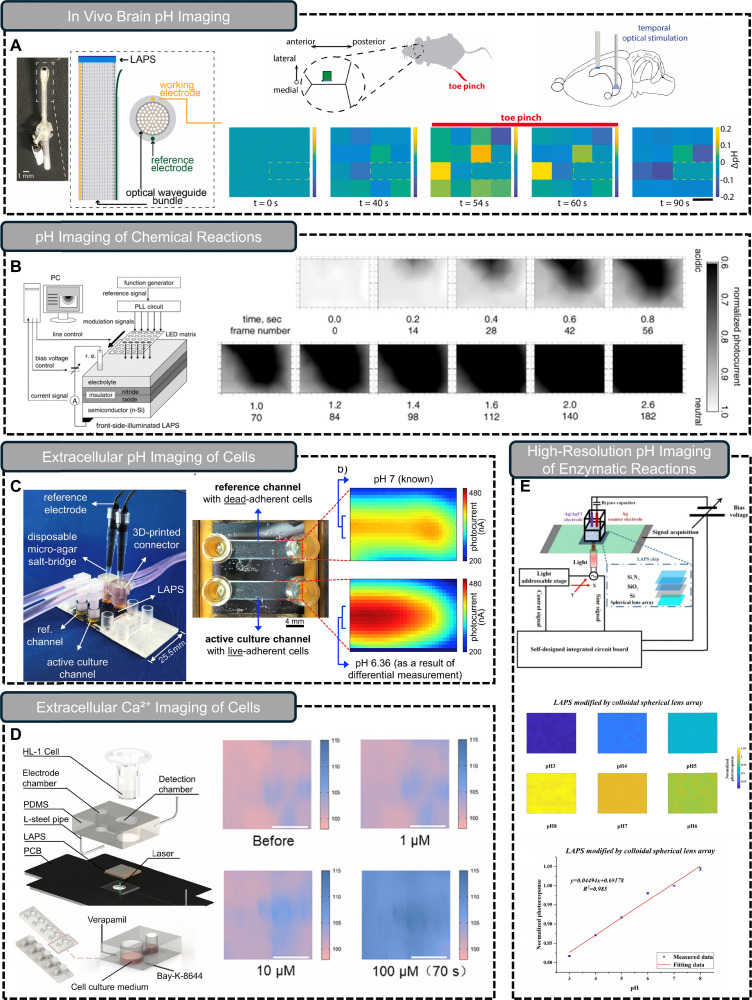
Representative applications of LAPS for chemical and biological imaging. (A) *In vivo* brain pH imaging demonstrating stimulus-evoked extracellular pH changes. Reproduced with permission from ref. 72. Copyright 2021 Elsevier. (B) Real-time imaging of dynamic pH changes during chemical reactions using frequency-division multiplexed LAPS. Reproduced with permission from ref. 59. Copyright 2013 Elsevier. (C) Extracellular acidification imaging of adherent CHO–K1 cells in microfluidic channels. Reproduced with permission from ref. 75. Copyright 2020 Elsevier. (D) Imaging of extracellular Ca^2+^ dynamics in living cells under pharmacological modulation. Reproduced with permission from ref. 86. Copyright 2025 Elsevier. (E) High-resolution spatiotemporal pH imaging enabled by a polystyrene colloidal spherical lens array. Reproduced with permission from ref. 73. Copyright 2024 Elsevier.

Monitoring brain pH is important because extracellular pH fluctuations are closely associated with neuronal activity, metabolic processes, and pathological conditions such as ischemia and seizures.^104–106^ In the work shown in Fig. 5A, LAPS is applied for *in vivo* pH imaging in the brain by integrating a Si_3_N_4_-based sensing chip with an optical fiber-guided illumination system, enabling localized light addressing within brain tissue.^72^ A toe-pinch stimulation is applied and the results showed that this stimulation led to measurable and dynamic changes in local brain pH, demonstrating the capability of LAPS to capture stimulus-evoked neurochemical responses with spatial resolution. In addition, similar extracellular pH monitoring is applied to cells for cell viability monitoring.

Fig. 5B demonstrates a LAPS system that utilizes FDM to achieve fast chemical imaging.^59^ The system successfully captures dynamic pH changes induced by injecting acid into solution, showing the spatiotemporal evolution of the chemical reaction, including diffusion and buffering effects. The imaging speed reaches 70 fps, allowing continuous observation of rapidly evolving chemical distributions. Furthermore, the authors suggest that a system with higher spatial resolution is under development. Other targets and chemical dynamics in other environments like microfluidic channels have also been studied.^31^ Such advancements highlight the potential of LAPS as a powerful tool for detailed analysis of chemical reaction dynamics and for applications in biological systems.

Monitoring extracellular chemical signals is essential for understanding cellular function, as many biological processes—including metabolism, ion transport, and signaling—are reflected through changes in the extracellular microenvironment.^75,86^ These signals can be probed either through general indicators, such as pH, or through specific targets, such as signaling ions. Fig. 5C demonstrates a LAPS-based system for imaging extracellular acidification, which serves as an important indicator of cellular metabolic activity. Cellular processes such as glycolysis and respiration lead to proton release, resulting in localized pH decreases near the cell surface. In this work, LAPS is integrated with a microfluidic platform and the extracellular acidification of adherent CHO–K1 cells inside microfluidic channels are visualized. Fig. 5D further demonstrates extracellular monitoring extended to ion-specific sensing of Ca^2+^ ions. The device employs a LAPS platform functionalized with an ion-selective membrane, with living cells cultured on the sensing surface to enable direct measurement of extracellular ion dynamics. The system produces spatially resolved Ca^2+^ imaging of cells, and further investigates changes in extracellular calcium concentration under pharmacological modulation, including calcium channel agonists and blockers. Other ions and other environments are also studied, like extracellular potassium ion, calcium ion in human serum and urine.^85,107^

Fig. 5E presents a LAPS system with enhanced spatiotemporal pH imaging performance.^73^ This improvement is achieved by introducing a polystyrene (PS) colloidal spherical lens array onto the sensor surface, which enhances light utilization and enables the device to operate at a higher modulation frequency. In the imaging of uniform pH solutions, the modified system exhibits a higher photoresponse amplitude and improved signal-to-noise ratio, along with a shift of the maximum photoresponse frequency from ∼10 kHz to 40 kHz, indicating enhanced signal quality and faster response. Finally, dynamic imaging of enzymatic reactions is demonstrated using a urease-modified LAPS, where the system successfully captures the real-time evolution of pH changes during urea hydrolysis, visualizing the formation and propagation of pH gradients over time.

Although LAPS has been applied to pH imaging, ion detection, enzyme-based sensing, aptamer-based recognition, and cellular measurements, further validation is still needed to support its reliability for biological and clinical applications. Some studies have begun to include application-specific validation. For example, an *in vivo* Si_3_N_4_-based pH LAPS probe evaluated pixel-to-pixel pH sensitivity, reference-electrode stability, calibration under physiologically relevant Cl^−^ concentrations, baseline-drift correction, and biological controls in rat hippocampus.^72^ However, such *in vivo* LAPS studies remain rare. Other studies have also incorporated useful validation steps. For instance, an alpha-fetoprotein (AFP) aptamer-functionalized LAPS evaluated human serum samples using a standard-addition method and compared the AFP level with chemiluminescence immunoassay.^62^ An extracellular Ca^2+^ LAPS platform for calcium-channel drug evaluation used Ca^2+^ calibration, background subtraction, cell-free drug controls, 2D/3D cell models, and fluorescence calcium imaging to support the interpretation of drug-induced Ca^2+^ responses.^86^ Nevertheless, these examples remain relatively limited and application-specific, and many LAPS studies still lack complete validation in complex biological matrices. Future work should place greater emphasis on comprehensive validation. Calibration should be performed in controlled *in vitro* solutions and biological matrices, followed by *ex vivo* tissue testing and *in vivo* validation when biomedical utility is claimed. Interference evaluation should include chemical interferents, electrical noise, mechanical deformation, optical intensity variation and distortion, and motion-induced signal changes. These effects may be reduced or separated using frequency-domain separation or mechanically or optically matched controls. Detection limit can be explored with simulated small signal, and repeatability should be tested with multiple batches of samples. Key metrics, including sensitivity, detection limit, response time and spatial resolution should be compared with established analytical or clinical methods. Such efforts will be important for moving LAPS from proof-of-concept demonstrations toward reliable biological and translational sensing platforms.

## 7. Applications of LAPS in electrophysiological detection

LAPS can also be applied to electrophysiological measurements, where extracellular bioelectrical activity modulates the interfacial potential of the EIS structure and is transduced into photocurrent variations under illumination. In contrast to chemical sensing, which originates from changes in surface charge or ion activity, electrophysiological detection captures dynamic extracellular potential, then linking photocurrent signals to cellular electrical activity. Current studies span multiple biological levels, including single-cell, organoid, and *in vivo* systems. The following section introduces representative works.

**Fig. 6:**
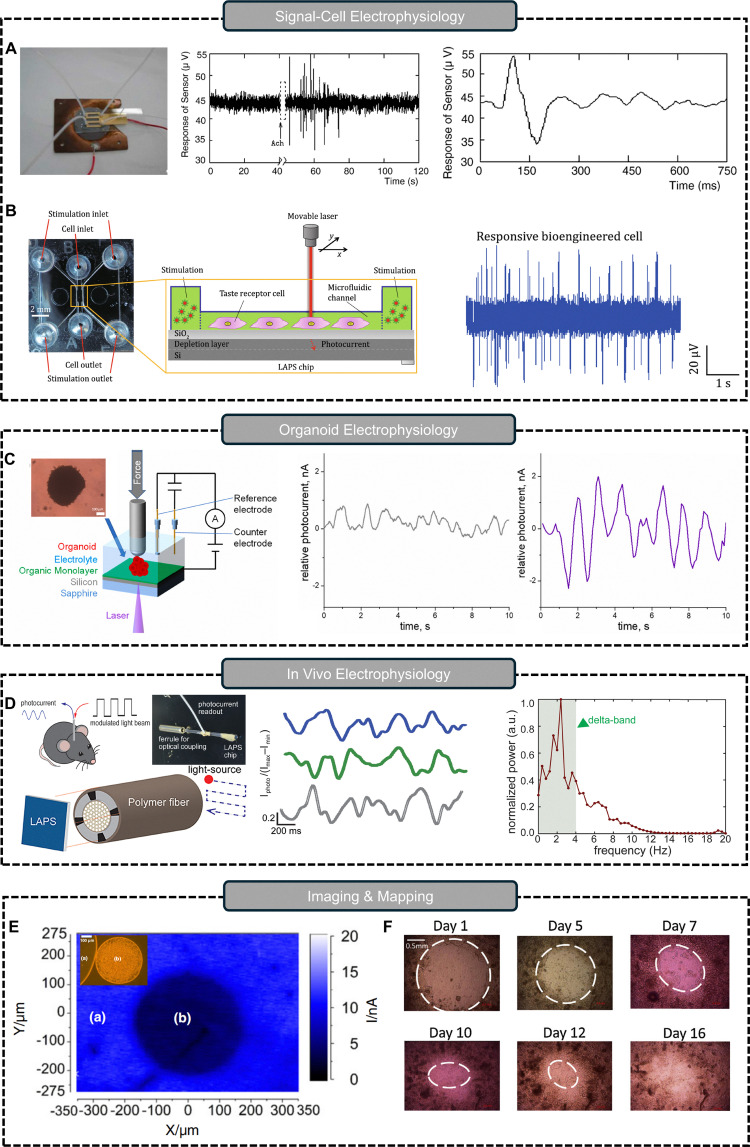
Applications of LAPS in detecting and imaging electrophysiological signals. (A) Single-cell electrophysiology using a cell-based LAPS platform, where extracellular potentials from individual neurons are recorded as photocurrent signals upon chemical stimulation. Reprinted with permission from ref. 39. Copyright 2005 Elsevier. (B) Single-cell electrophysiology using a microfluidic-integrated LAPS platform, enabling selective stimulation and extracellular recording of bioengineered taste receptor cells. Reprinted with permission from ref. 38. Copyright 2019 Springer Nature. (C) Organoid electrophysiology using a photoelectrochemical LAPS platform, detecting electrical activity from cardiomyocyte organoids with enhanced signal under controlled contact conditions. Reprinted with permission from ref. 111. Copyright 2023 Elsevier. (D) *In vivo* electrophysiology using a fiber-coupled LAPS device, enabling deep-brain recording of local field potentials and frequency-domain analysis. Reprinted with permission from ref. 40. Copyright 2020 PLOS ONE. (E) LAPS-based imaging of individual cells, enabling cell distribution through surface charge and impedance contrast. Reprinted with permission from ref. 113. Copyright 2016 Elsevier. (F) LAPS-based chemical imaging of cell layers, enabling spatiotemporal monitoring of defect formation and recovery processes. Reprinted with permission from ref. 35. Copyright 2016 Elsevier.

Fig. 6A and B illustrate LAPS-based electrophysiological measurements at the single-cell level, where localized light addressing enables spatially resolved extracellular recording of membrane potential dynamics. In such cases, excitable cells including neurons, cardiomyocytes, and specialized receptor cells modulate the local surface potential *via* ion fluxes associated with membrane activity, which is subsequently transduced into photocurrent variations under modulated illumination.^22,108–110^ For example, in Fig. 6A, a cell-based LAPS platform enables extracellular recordings from individual neurons. The device uses an n-type Si wafer with a thermally grown ∼30 nm SiO_2_ layer, and the backside is thinned to ∼100 µm to increase the sensitivity by shortening the travel distance of photocarriers, reducing recombination losses and limiting the lateral diffusion of the photocarriers.^40^ Coating with Al forms the ohmic contact. Cortical neurons are cultured directly on the sensing surface within a polydimethylsiloxane (PDMS) microchamber. A focused light spot selectively addresses a single cell from the top and enables localized measurement. Upon acetylcholine stimulation (1–10 µg mL^−1^), the sensor response shows clear signal modulation, and the enlarged trace exhibits spike-like transients corresponding to extracellular action potentials. The recorded signals display a characteristic waveform with a fast-rising peak followed by a phase of ∼100–150 ms and an amplitude of ∼7–12 µV, consistent with extracellular action potential signals reported using traditional field-effect transistor (FET)-based recordings. In Fig. 6B, integration of LAPS with a microfluidic platform enables single-cell functional assays.^38^ The device uses a Si-based structure with a thermally grown SiO_2_ insulting layer (∼30 nm) and an additional Si_3_N_4_ layer (∼60 nm) as an ion barrier, while the backside is thinned to ∼100 µm and coated with Al to form the contact. A microfluidic chip with a central cell chamber and side stimulation reservoirs is mounted on the LAPS surface to enable controlled delivery of chemical stimuli.

During measurement, bioengineered taste receptor cells are cultured in the microchannel, and a focused light beam from the top side selectively addresses individual cells. Chemical stimulation introduced through the microfluidic channel induces membrane potential changes and generates photocurrent fluctuations. As shown in the figure, regarding a specific taste-related chemical (*e.g.* denatonium for bitter), responsive cells exhibit dense spike trains upon stimulation, whereas non-responsive cells show only weak activity. The firing rate of responsive cells (19.2 ± 3.10 Hz) is significantly higher than that of non-responsive cells (5.6 ± 1.30 Hz), enabling clear identification of functional cells based on their electrophysiological response.

Moreover, it should be noted that photocurrent signals in LAPS-based electrophysiological measurements can contain multiple contributions, including extracellular potential changes, cell–surface coupling, optical modulation, and mechanical deformation. As demonstrated in the cardiomyocyte-LAPS study by Parak *et al.*, mechanical deformation and optical modulation can strongly affect photocurrent signals and obscure true extracellular potentials.^23^ Therefore, reliable interpretation requires proper signal processing and artifact control. Several studies discussed in this section employ different strategies to improve signal reliability and reduce possible artifacts. For example, wavelet-transform de-noising removes baseline drift and improves the signal-to-noise ratio in PC12-cell LAPS recordings,^108^ while lock-in detection, low-pass filtering, and fast Fourier transform (FFT) analysis extract local field potential signals from fiber-coupled *in vivo* recordings.^40^ More recently, Jacques *et al.* further explored strategies to minimize optical and mechanical artifacts in cardiomyocyte-organoid LAPS measurements.^111^ As a result, backside illumination avoided direct light interaction with cells, controlled contact force improved cell-sensor coupling and reduced the cell-surface gap, and blebbistatin treatment suppressed cardiomyocyte contraction to verify that the remaining photocurrent signals mainly originated from electrophysiological activity rather than mechanical beating.^111^ These efforts provide useful examples for separating true electrophysiological signals from possible artifacts. However, systematic studies on artifact separation in LAPS-based electrophysiological measurements remain limited and require further investigation with standardized controls and direct comparison with established electrophysiological methods.

Fig. 6C illustrates LAPS-based electrophysiological measurements at the organoid level, where collective extracellular potential dynamics from multicellular assemblies are monitored, enabling the investigation of functional activity beyond single-cell resolution.^111^ In this case, a LAPS platform enables electrophysiological recording from cardiomyocyte organoids. The device uses SOS structure with a 0.5 µm Si layer supported on a 475 µm sapphire substrate, and an ultrathin alkyne-terminated SAM serves as the insulating layer. Backside illumination through the transparent substrate enables localized photoexcitation. During measurement, a cardiomyocyte organoid is placed on the sensing surface, and a controlled contact force (∼0.02 N) is applied to improve electrical coupling. A focused laser excites the Si layer, and the resulting photocurrent reflects the electrical activity of the organoid. As shown in the figure, the recorded photocurrent exhibits periodic oscillations corresponding to spontaneous action potential. Increased contact enhances the signal amplitude by more than 4.2 times and improves waveform clarity, corresponding to an average action potential amplitude of ∼8.1 mV, thereby enabling stable electrophysiological recording from the organoid.

Fig. 6D illustrates LAPS-based electrophysiological measurements at the *in vivo* level, where neural activity is recorded directly from living brain tissue, enabling real-time monitoring of physiological signals under native biological conditions. A fiber-coupled LAPS platform enables label-free electrophysiological recording in deep brain regions.^40^ The device uses a miniaturized Si-based LAPS chip prepared from boron-doped silicon wafers with 50 nm SiO_2_ as a waterproof layer and 50 nm Si_3_N_4_ as an ion barrier layer. The silicon thickness is reduced from 200 µm to below 100 µm, and thin Ti/Au layers are formed on the backside to provide ohmic contact while maintaining optical transparency. During measurement, the LAPS chip is coupled to a polymer fiber and implanted into the mouse hippocampus, where modulated light is delivered through the fiber to generate photocurrent. As shown in the figure, representative photocurrent traces reveal local field potentials recorded in the hippocampus of anesthetized mice. Power spectral analysis further shows a clear delta-band component, demonstrating the feasibility of *in vivo* deep-brain recording with the fiber-coupled LAPS device.

Besides electrophysiology, LAPS can also be applied to spatial imaging and mapping of cellular properties, including surface charge distribution,^112^ local impedance variations,^113^ cellular morphology contrast,^114^ and dynamic changes in cell-layer integrity.^35^Fig. 6E and F demonstrate LAPS-based impedance imaging. In Fig. 6E, LAPS detects the spatial distribution of individual cells based on their surface charge and local impedance.^113^ A LAPS platform based on SOS structure with a ∼1 µm Si layer supported on a 475 µm sapphire substrate serves as the imaging sensor, where an SAM serves as the insulating layer. A focused and modulated laser scans across the surface, and the local photocurrent generates a spatial map. Immobilized yeast cells produce clear photocurrent contrast due to their negative surface charge and increased local impedance, enabling label-free imaging with micrometer-scale resolution. In Fig. 6F, LAPS monitors the spatial variation of cell-layer integrity during the recovery process.^35^ A chemical imaging sensor employs an n-type Si substrate with double insulating layers of SiO_2_/Si_3_N_4_ (50 nm/50 nm). The cultured cell layer on a permeable membrane contacts the sensing surface, and a scanning laser generates photocurrent images. As shown in the figure, a high photocurrent region emerges at the defect center, and the high-signal area gradually decreases during the recovery process. The defect shrinks over time and completely heals within approximately 12–15 days, demonstrating the capability of LAPS for dynamic monitoring of cell-layer integrity. Representative studies on LAPS-based electrophysiological detection are summarized in Table 3, including their biological targets, sensing interfaces, demonstrated applications, and laser systems.

**Table 3:** Summary of representative LAPS studies for electrophysiological detection

Year	Target	Sensing material	Operating condition	Application demonstrated	Laser system	Limitations	Ref.
1998	Membrane potential	Si_3_N_4_/SiO_2_ on Si	L-15 culture medium	Extracellular electrophysiological recording of neural cells; mapping membrane potential dynamics	Semiconductor laser (833 nm)	Sampling speed limits nerve pulse detection	22
2005	Extracellular action potential	SiO_2_	DMEM	Single-cell extracellular action potential recording; drug stimulation (acetylcholine) response monitoring in cultured rat cortical neurons	He–Ne laser (543.5 nm)	Sensitivity decreases after repeated cycles	39
2010	PC12 cell (Extracellular potential)	SiO_2_	DMEM	Extracellular potential recording with signal processing (wavelet denoising) for neural activity monitoring	He–Ne laser (543.5 nm)	Low signal/noise ratio and baseline drift require wavelet denoising	108
2008	Taste receptor cells (extracellular membrane potential)	SiO_2_	DMEM	Detection of extracellular potentials under chemical stimulation (taste transduction study)	He–Ne laser (543.5 nm)	On-chip immunostaining is limited by opaque LAPS substrate	109
2018	Taste bud cells (extracellular membrane potential)	SiO_2_/ATP sensitive layer	Tyrode's solution	Dual-functional extracellular recording and stimulation response monitoring for bitter signal transduction	He–Ne laser (543.5 nm)	Regeneration of ATP-aptamer sensing capability and broader tastant/cell validation are needed	110
2018	Bioengineered taste receptor cells (extracellular membrane potential)	SiO_2_	DMEM	Identification of responsive cells under bitter stimulation (denatonium); single-cell analysis in microfluidic environment	Semiconductor laser (543.5 nm)	Demonstrated only with T2R4-engineered cells and denatonium; broader validation is beneficial	38
2023	Cardiomyocyte action potentials	SAM-modified Si on sapphire	DMEM	Photoelectrochemical imaging of cardiomyocyte action potentials	Laser (405 nm)	Requires controlled contact force; excessive force damages organoids	111
2020	Extracellular potential	SiO_2_	Saline	Label-free electrophysiological recording in mouse hippocampus (*in vivo*); spatially resolved neural activity mapping *via* fiber-coupled LAPS	Diode-pumped solid-state laser (473 nm)	Initial *in vivo* test used single-pixel addressing	40
2016	Yeast cells	SOS with 1,8-nonadiyne monolayer	PBS	Label-free imaging of yeast cells *via* local surface charge and impedance	Diode laser (405 nm)	Single-layer/few cells are not clearly detected	113
2016	Cultured cell layer defect	Si_3_N_4_/SiO_2_ on Si	DMEM	Impedance imaging of artificial defects in a cultured cell layer; monitoring defect recovery/wound-healing over time	Semiconductor laser (830 nm)	Requires reproducible membrane–sensor spacing	35

## 8. Conclusions and outlook

This review summarizes recent advances in the development of LAPS for the detection of biochemical and biophysical signals, with particular emphasis on its potential for spatially resolved applications in biological systems. This concept has evolved over the past few decades, spanning a broad range of biological models through interdisciplinary efforts in semiconductor physics, optoelectronics, biosensing, and electrical engineering. By properly designing the sensing interfaces using corresponding bio-recognition elements (*e.g.*, ion-selective membranes, aptamers, enzymes, antibodies) to induce responses in surface potential, this sensing strategy is compatible with the mapping of a variety of analytes (*e.g.*, ions, hormones, growth factors, metabolites), making the system highly versatile for different application scenarios. As discussed in the preceding sections, examples of demonstrated and potential future applications highly relevant to biomedical research and human health include, but are not limited to: (1) pH changes in ischemic tissues due to lack of O_2_ leading to glycolysis,^20^ (2) extracellular Na^+^ and K^+^ concentrations in brain tissues as biomarkers for secondary traumatic brain injury,^21^ (3) distribution of biomolecules in tissues such as cytokines, chemokines and/or growth factors associated with wound healing and regenerative process. In addition to detecting chemical biomarkers, this voltage-detection sensing mechanism is compatible with detecting membrane potential of cells, such as neurons^22^ and cardiac myocytes,^23^ as showcased in pioneering studies.

Here, we also summarize the limitations of current LAPS systems: despite significant success in achieving sensing performance in cell cultures, organoids, and biotissues, applications in *in vivo* models remain underexplored compared to conventional wired sensing platforms. As described in Fig. 2, conventional LAPS platforms based on thick, rigid silicon wafers suffer from limited flexibility, low voltage sensitivity, and poor spatial resolution due to long carrier diffusion lengths, while alternative substrates (*e.g.*, SOI, SOS, and III–V materials) present challenges in optical compatibility, fabrication, stability, and biocompatibility. Reproducibility and scalability are also important considerations for LAPS fabrication. Among the device architectures discussed above, Si-based LAPS remains the most mature platform because Si processing is compatible with established complementary metal-oxide-semiconductor (CMOS) strategies. This maturity provides advantages in dielectric formation, device processing, and fabrication reproducibility. Meanwhile, alternative architectures, such as SOS and direct-bandgap semiconductor-based LAPS, have been explored to improve spatial resolution or device performance. However, these approaches also introduce additional fabrication challenges. In SOS structures, thermal mismatch between Si and sapphire during oxide growth can induce cracking of the thin semiconductor layer. For direct-bandgap semiconductor materials, the formation of high-quality insulating layers remains challenging and can introduce interface defects, charge trapping, leakage current, and signal instability. Moreover, it is also noted that thick bulk Si wafers restrict spatial resolution because photogenerated carriers laterally diffuse through the semiconductor before reaching the space-charge region, whereas rigid planar substrates limit conformal integration with soft biological interfaces. Mechanical thinning can reduce carrier diffusion, but grinding produces fragile ultrathin wafers and lacks the process standardization needed for reliable batch-to-batch control over thickness uniformity, surface quality, and device yield. All of these considerations suggest that future LAPS fabrication should move toward more standardized micro/nanofabrication processes and flexible device architectures to reduce batch-to-batch variation and improve scalability and compatibility with bio-interfaces.

In addition, flexible device architectures are also highly desirable for shape-adaptable deployment on biological tissues to monitor dynamic processes. Future efforts should also address optical challenges associated with operation on curvilinear surfaces. In particular, nonuniform light intensity arising from variations in pixel location, tilt angle, and depth necessitates the incorporation of adaptive refocusing mechanisms to ensure accurate and uniform pixel addressing. In addition, systematic trade-off analyses of the optical depth of focus are needed to balance spatial resolution, refocusing frequency, and sensitivity to surface curvature. Finally, establishing standardized calibration protocols for devices operating on curvilinear geometries will be critical, enabling accurate quantification of signals across tissues with varying bending radii and ensuring reliable performance in diverse anatomical environments.

For *in vivo* applications, a critical challenge is achieving addressing beyond the tissue surface, which currently limits broader applicability. While light replaces conventional wiring to enable localized addressing, future efforts should focus on expanding this capability to subsurface regions. The light source itself, although an additional module, offers strong potential for miniaturization and integration. Once light-addressable operation is established, alternative formats such as micro-LED arrays,^115^ implantable waveguides (*e.g.*, optical fibers),^116^ and tissue-penetrating deep-red or near-infrared light^117^ can further enhance usability and depth accessibility. Ultimately, these developments should aim to achieve cellular-scale spatial resolution for precise *in vivo* interrogation.

Beyond these mechanical and optical challenges, real-world biomedical implementation also requires careful consideration of biocompatibility, chronic stability, and system integration. For long-term implantation or repeated *in vivo* use, LAPS devices must minimize inflammatory responses, semiconductor or dielectric degradation, and drift of the functional sensing interface. Stable encapsulation strategies are also needed to protect non-sensing regions while preserving the chemical accessibility of the active sensing area. In addition, practical biomedical use will require portable readout systems that integrate compact light modulation, low-noise photocurrent amplification, automated scanning or pixel addressing, wireless data transmission, and real-time signal processing. Therefore, future LAPS development should address mechanical compliance, optical access, biological stability, and portable instrumentation together rather than treating them as separate engineering problems.

Overall, we hope that the discussions presented here will inform the transition of LAPS from benchtop evaluation systems to viable *in vivo* applications. More broadly, site-specific light–matter interactions offer a versatile platform for energy and information transfer across diverse systems, potentially inspiring applications beyond the current scope, including electrochemical processes and biological modulation. Continued interdisciplinary efforts will be essential to advancing this field and realizing impactful outcomes in the future.

## Author contributions

Yizhen Jia: visualization, writing – original draft. Shulin Chen: visualization, writing – original draft. Jinghua Li: supervision, writing, editing, and funding acquisition.

## Conflicts of interest

There are no conflicts to declare.

## Data Availability

This study does not generate any new datasets. All data analysed are from publicly available sources, as cited in the manuscript.
